# Radiation-induced Alopecia

**DOI:** 10.4103/0974-7753.77528

**Published:** 2010

**Authors:** Syed Yousuf Ali, Gurcharan Singh

**Affiliations:** Department of Dermatology and STD, Shadan Institute of Medical Sciences, Peerancheru, Hyderabad, Andhra Pradesh, India; 1 Department of Dermatology and STD, Sri Devaraj Urs Medical College, Kolar, Karnataka, India

Sir,

Radiotherapy is a common modality in cancer treatment and more than 50% of affected patients will eventually receive some form of radiotherapy as definite, preoperative, postoperative or palliative treatment.[1]

Radiotherapy can result in certain inevitable side effects, radiation-induced cutaneous side effects that include acute and chronic radiodermatitis[2] and systemic side effects.[3] Skin may be injured as an ‘innocent bystander’ and develop profound alterations on functional, gross and molecular levels.[4]

**Figure 1:**
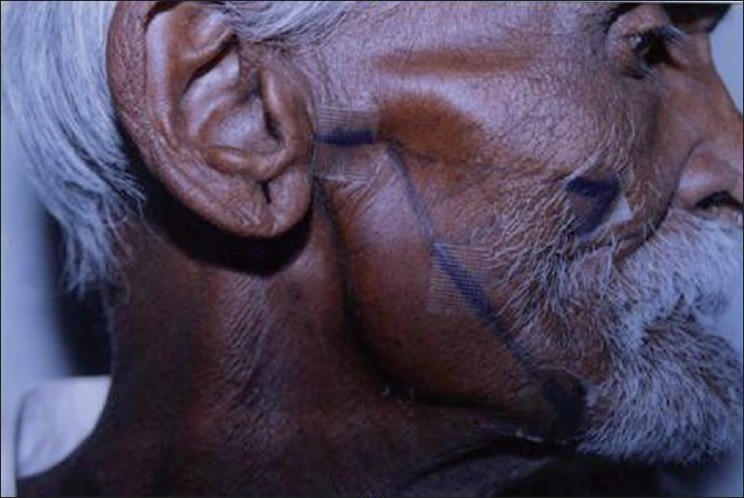
Radiation-induced alopecia

A 65-year-old man presented with a history of hair loss in the beard area while receiving radiotherapy for oropharyngeal carcinoma. Hair loss over the beard area was noted two weeks after the initiation of radiotherapy. There was no history of chemotherapy or surgery done for the tumor. Physical examination revealed 10×8 cm irregular patch of near total hair loss of the beard region [Figure 1]. The beard area was normal and exclamation-mark hairs were not visible; results of the pull test were normal. Excluding the diagnosis of alopecia areata may be difficult because the bald patch is devoid of inflammatory signs and hair loss is characterized by dystrophic hair.

It was not long before that the physiological effects of radiation were noticed. In April 1896, Daniel described epilation and a serious skin reaction after prolonged exposures; other reports soon followed, the realization that the large doses of X-rays produced harmful effects on the skin suggested that beneficial effects on skin diseases might be obtained with lower doses. In 1896, Freund of Vienna observed epilation by X-ray therapy of a large hairy nevus and followed this by treating various inflammatory diseases, including eczema, psoriasis and ringworm, with X-rays.[5]

Irradiation-induced epilation is due to high susceptibility of anagen follicles to radiation. Loss of dystrophic hairs (anagen effluvium) due to acute damage to actively dividing matrix cells of anagen follicles is followed by telogen shedding due to premature catagen entry of follicles in late anagen.[6] 3GY produces complete, reversible anagen alopecia; permanent alopecia begins to occur at 5GY.[7,8] Complete hair regrowth generally occurs 2−4 months after irradiation in reversible type of radiation-induced alopecia.[6] Post-radiotherapy and permanent alopecia can be addressed by reconstructive surgery.[9] Use of nitroxides tempol and tempo, vitamin D3 and 16, 16-dimethyl prostaglandin E2 (PGE2) prior to radiation have been shown to protect against radiation-induced alopecia.[10–12]

The present case is being reported for the awareness of the radiation-induced alopecia as radiotherapy is the commonly used modality for the treatment of head and neck carcinomas.
